# Anesthesiology management for the transcatheter aortic valve implantation

**DOI:** 10.1186/1749-8090-8-S1-O317

**Published:** 2013-09-11

**Authors:** N Bradic, I Husedzinovic, Z Sutlic, D Unic

**Affiliations:** 1 Clinic of Anesthesiology, Reanimatology and Intensive Medicine, University Hospital Dubrava, Zagreb, Croatia; 2 Clinic of Surgery, Department of Cardiac and Transplant Surgery, University Hospital Dubrava, Zagreb, Croatia

## Background

The aim is to present the results of the two-years’ experience in the anesthetic management for percutaneous transcatheter aortic valve implantation (TAVI) procedures.

## Methods

All procedures were performed in period between March 2011 and May 2013.In that period, 26 procedures performed in patients with severe aortic stenosis: of total, sixteen patents received Medtronic/CORE valve. Ten patients received Edwards/Sapien Aortic Bioprosthesis, of which, one implanted by transaortic way. The average age was 78.43 years. In premedication, all received midazolam 5 mg and clopidogrel 150 mg. All procedures were performed in general anesthesia using sevoflurane, small doses of i.v. fentanyl and rocuronium at the start of the procedure. Anesthesia maintained with sevoflurane up to the end of procedure. Rapid ventricular pacing achieved with transvenous temporary pacemaker. For evaluation of aortic valve, aortic root, left ventricular outflow tract pre and after procedure, in all patients transesophageal echocardiography (TEE) was used.

## Results

The valve was successfully implanted in 25 patients; one valve could not be positioned adequately. In seven patients, TEE evaluation showed needing for valve re-expanding due to perivalvular leak. Four patients needed short usage of norepinephrine due to hemodynamic instability. 21 patients were extubated in hybrid operating room and transferred to ICU for the next 24 hours. Early postoperative complications included new left bundle branch block in one patient, one stroke and one heart failure in further two patients. All patients discharged from hospital within 7 to 10 days after the procedure in good conditions.

## Conclusion

TAVI procedure has successful outcome in selected high-risk patients. General anesthesia is optional because it provides better hemodynamic stability, it is more comfortable for the patient, and finally, facilitates better conditions for precise and extremely important TEE estimations during procedure.

